# State of the Art, Unresolved Issues, and Future Research Directions in the Fight against Hepatitis C Virus: Perspectives for Screening, Diagnostics of Resistances, and Immunization

**DOI:** 10.1155/2016/1412840

**Published:** 2016-10-24

**Authors:** Cecilia Trucchi, Andrea Orsi, Cristiano Alicino, Laura Sticchi, Giancarlo Icardi, Filippo Ansaldi

**Affiliations:** ^1^Department of Health Sciences, University of Genoa, 16132 Genoa, Italy; ^2^IRCCS AOU San Martino-IST, 16132 Genoa, Italy

## Abstract

Hepatitis C virus (HCV) still represents a major public health threat, with a dramatic burden from both epidemiological and clinical points of view. New generation of direct-acting antiviral agents (DAAs) has been recently introduced in clinical practice promising to cure HCV and to overcome the issues related to the interferon-based therapies. However, the emergence of drug resistance and the suboptimal activity of DAAs therapies against diverse HCV genotypes have been observed, determining treatment failure and hampering an effective control of HCV spread worldwide. Moreover, these treatments remain poorly accessible, particularly in low-income countries. Finally, effective screening strategy is crucial to early identifying and treating all HCV chronically infected patients. For all these reasons, even though new drugs may contribute to impacting HCV spread worldwide a preventive HCV vaccine remains a cornerstone in the road to significantly reduce the HCV spread globally, with the ultimate goal of its eradication. Advances in molecular vaccinology, together with a strong financial, political, and societal support, will enable reaching this fundamental success in the coming years. In this comprehensive review, the state of the art about these major topics in the fight against HCV and the future of research in these fields are discussed.

## 1. Introduction

Among infectious diseases, hepatitis C virus (HCV) still represents a major public health threat, with a dramatic burden from both epidemiological and clinical points of view. Chronically infected individuals are estimated to reach 150–170 million worldwide and estimates of incidence, performed in the United States by the Center for disease control and prevention (CDC), reported nearly 30,000 new HCV infections in 2013 [1, 2].

Although HCV infection is characterized by a global diffusion, its prevalence greatly differs according to geographic area [3, 4]. Central Asia, Eastern Europe, the Midwest of North Africa region, and Central and Western Sub-Saharan Africa present high HCV prevalence rates, with figures ranging between 3.1% and 5.4%; regions with intermediate prevalence rates are Southern Sub-Saharan Africa, Central Europe, Australia, and Latin America, with values between 1% and 1.4%; low prevalence is found in Oceania (0.1%), Caribbean (0.8%), and Western Europe (0.9%) [3].

After acute infection, 75% of infected subjects become chronically infected and approximately 20% of this population develops liver cirrhosis during the two decades after infection if left untreated [5, 6]. However, since in most cases acute infection was asymptomatic, most HCV infections are clinically silent until the disease reaches a late stage: HCV was estimated to cause 25% of all cases of liver cirrhosis and cancer worldwide and to account for more than 500,000 deaths per year [7].

In recent years, substantial advances have been made to understand HCV biology and to develop a new generation of effective direct-acting antiviral agents (DAAs) able to cure HCV. However, several challenges hamper an effective control of HCV spread worldwide. In fact, the emergence of drug resistance and the suboptimal activity of these therapies against diverse HCV genotypes have been observed and have been associated with treatment failure. Moreover, the high costs of these drugs and the high prevalence of HCV-infected individuals, especially in low-income countries, jeopardized the affordability for the healthcare system to treat all infected patients in developed countries and, even more, in developing countries [8, 9]. Finally, effective screening strategy is required to early identify and treat all HCV chronically infected patients thus limiting the infection transmission risk as well as the progression to cirrhosis or hepatocellular carcinoma and reducing the healthcare costs [10, 11]. For all these reasons, a preventive HCV vaccine remains a cornerstone in the road to significantly reduce the HCV spread globally.

This comprehensive review summarized the state of the art about three major unresolved issues in the fight against HCV: which are the perspectives for the universal screening of HCV? Do we need DAAs resistance testing in the future? How close is an effective preventive HCV vaccine?

## 2. Which Are the Perspectives for the Universal Screening of HCV?

The rate of underdetection of HCV infection is still relevant because of clinical, educational, technical, organizational, and economic issues. In fact, recent estimates suggest that most of people with HCV remain undiagnosed or unaware of their HCV infection [12, 13].

Another criticism is represented by the difficulty in early diagnosing HCV infection. Indeed, few people are diagnosed during the acute phase because it is usually asymptomatic [14]. Furthermore, the 55–85% of persons who do not spontaneously clear the virus within 6 months develop chronic infection and remain asymptomatic for decades after infection, during which infection may be transmitted to other persons. Chronically infected patients usually become symptomatic when the HCV-induced liver damage is advanced and the therapy may be contraindicated [14]. The risk of late diagnosis is associated also with the limited access to HCV testing in many countries where HCV prevalence is high, such as African and Central-East Asian countries [15].

Therefore, it is crucial to implement the most sensitive and specific approaches to diagnose chronic HCV infection before the development of liver damage and to assure the linkage to care of infected patients [16].

### 2.1. Screening Tests for HCV Infection

A testing strategy for HCV infections characterized by high sensitivity and specificity should be established.

The WHO recommends offering the HCV serology test to individuals belonging to population with high HCV prevalence or who have a history of HCV risk exposure/behavior [14]. Given that HCV antibodies can be detected two months following the infection and are also detectable in patients who have cleared the virus, a positive result for antibodies against viral proteins (anti-HCV) should be followed by a nucleic acid testing using a reverse transcription polymerase chain reaction (RT-PCR) for the detection of HCV RNA and to confirm chronic HCV infection [14, 17]. Molecular testing is more technically demanding than the serology but it guarantees positive results already after few weeks following the exposure.

Among serologic screening test, the enzyme immunoassays (EIAs) are generally used [18]. EIAs are characterized by high sensitivity and specificity, fast processing, high reliability, relatively low costs, and the possibility of automation, useful for large volume testing [19–21]. In particular, the third generation test of EIAs is available [22] and has an estimated sensitivity and specificity of 98.9% and 100%, respectively, in patients with chronic liver disease [23]. However, the serological window period of this assay is generally more than 40 days [24] and it should not be used in infants younger than 18 months because of the possible reaction with maternal antibody [25].

Recently, the possibility of a single serologic assay for the detection of HCV active infection has become available. This test is based on the identification of the HCV core antigen (HCVAg) that is detectable in acute infection almost at the same time as HCV RNA [26] and persists during all phases of infection [27], indicating its potential use as a less expensive technique than molecular assays.

In consideration of improving the testing access, some countries such as US [28] and France [29] validated the use of Point of Care (POC) not only for HIV but also for HCV infection screening. Although rapid tests have lower sensitivity than standard serological tests and require a specific organization to allow traceability of results, they are characterized by relevant advantages such as the minimal equipment required, the ease of obtaining the samples and of performing the test, and the short time to obtain the results [30, 31]. However a recent meta-analysis that compared seven POC demonstrated that OraQuick had the highest test sensitivity and specificity and showed better performance than a third generation enzyme immunoassay in seroconversion panels [32]. The availability of POC rapid tests represents an opportunity to broaden the screening strategies to people outside the healthcare structures [11], thus helping the achievement of individuals at highest risk, such as people who inject drugs, the homeless, and the incarcerated.

### 2.2. Identifying Patients with HCV

The variability of the risk factors and history of behaviors linked to HCV infection, depending on the geographical setting and population studied, make the identification of the target population for screening policies challenging worldwide.

In many high-income countries HCV screening recommendations are targeted to subjects with a history of HCV risk exposure or behavior and certain medical conditions and who belong to a population of known high HCV prevalence [33, 34]. In particular, at risk populations include those who have received medical procedures such as hemodialysis or dental interventions in healthcare facilities with inadequate infection control practices, persons who have received blood transfusions, an organ transplant, or tissue graft prior to the time when HCV serological testing of blood donors was introduced or in countries where it is not routinely performed, subjects who inject drugs or who have used intranasal drugs, persons who have received tattoos, body piercing, or scarification procedures in settings where infection control practices are substandard, children born to mothers infected with HCV, healthcare workers who sustained a needle stick or mucosal splash exposure from a patient with HCV, and patients with HIV infection who have unprotected sex with men, prisoners, and previously incarcerated persons (Class I, Level B recommendation) [10, 16, 35].

In 2012, the Center of Disease Control and Prevention (CDC) and the US Preventive Services Task Force (USPSTF) extended the HCV screening recommendation to all persons born from 1945 through 1965, without prior ascertainment of HCV risk factors (Class I, Level B recommendation) [35–37]. The rationale of this recommendation is based on the evidence that the risk-based strategy alone failed to identify more than 50% of HCV infections because of healthcare provider limitations in ascertaining risk factor information [38, 39] and due to patients' underreporting of their own risk behaviors (e.g., injecting drug use) or exposure (e.g., iatrogenic infection). Furthermore, persons in the 1945 to 1965 birth cohort accounted for about 75% of all HCV infections, with a five times higher prevalence (3.25%) than other persons [10, 37]. A recent retrospective review demonstrated that 68% of persons with HCV infection would have been screened through a birth cohort approach testing, while just 27% would have been identified with the risk-based strategy [40]. The cost-effectiveness of one-time birth cohort testing is comparable to that of current risk-based screening strategies [36]. Nevertheless, the implementation birth cohort screening is challenging [10]. The inclusion of queries and reminder flags among the electronic medical record (EMR) could sensitize physicians [41]. However, various types of EMR exist in USA and many reminders requiring time to be addressed are triggered during each visit [10]. Furthermore, the identification of the best setting to implement the screening of birth cohort represents a further issue. Available evidences suggest that the hospital setting allows reaching better results than the outpatients' visits such as routine colon cancer screening colonoscopy [42–45].

In Japan, where the overall prevalence of HCV infection is comparable with the USA, but different transmission patterns determined different age-specific prevalence, the national screening for HCV in both the high-risk group and the general population from ages 40 to 70 started at 2002 and has demonstrated to be cost-effective in containing the epidemic [46, 47].

In Europe, accurate estimates of HCV incidence and prevalence are not available; however prevalence estimates vary from 0.4% (Austria, Cyprus, Germany, Denmark, France, and United Kingdom) to 1.5% (Israel and Italy) in Western Europe and from 0.7% (Czech Republic) to 4.5% (Moldova) in Central and Eastern Europe [46]. HCV screening programs are implemented for organ and blood donors as well as for patients undergoing hemodialysis, but not for risk groups such as injecting drug users, who currently represent the leading cause of transmission across Europe [48, 49]. Furthermore, the rising issue of fluid immigration patterns from countries with high prevalence such as Egypt has not been addressed yet [50].

Developing nations, where the population-based prevalence of anti-HCV may reach as high as 11% (Mongolia) to 15% (Egypt), are less likely to be able to afford screening [46]. Many of these countries are afflicted by political and social unrest that may influence risk factors of HCV spread, such as trauma, use of intravenous drugs, poor water and electric supply, overcrowding, and lack of financial resources and infrastructure [46].

Considering the existing barriers that limit the implementation of screening strategies such as low provider knowledge about HCV and its related risk factors [13, 51–54], the WHO Guidelines Development Group conducted a systematic review to find the evidences about the most effective strategies to promote HCV testing [16]. Sensitizing the practitioners about the importance of early HCV diagnosis through in-service training sessions or mailed information, provision of additional clinic staff, routine offer of testing to all patients, or placing reminders in medical records resulted more effectively in increasing uptake of testing, detecting HCV antibody-positive cases, and the number of attendances and referral to specialist care than media-/information-based targeted approaches (e.g., invitations to information sessions for care providers, leaflets, and posters).

### 2.3. Recommendations for Frequency of HCV Screening Testing

CDC and the US Preventive Services Task Force (USPSTF) recommend a one-time HCV test in asymptomatic persons belonging to the 1945 to 1965 birth cohort and subjects with exposures, behaviors, and conditions that increase risk for HCV infection.

As regards the correct periodicity of testing persons at risk for ongoing exposure to HCV, evidence is lacking. Thus, physicians should determine the frequency of testing on the basis of the risk of reinfection. Owing to the high incidence of HCV infection among HIV-infected men who have unprotected sex with men and subjects who inject drugs [55–60], the HCV Guidance by the Infectious Diseases Society of America (IDSA) and the American Association for the study of liver diseases (AASLD) recommended that at least annual HCV testing is in these subgroups [35].

### 2.4. Linkage to Care

In order to enter the patients in the cascade of care and thus provide them with the so-called “continuum of care,” the detection of HCV-infected patients should be followed by linkage to care via a physician referral with expertise in evaluation and treatment HCV infection [10, 61]. However, available evidences show that many gaps between the HCV diagnosis and care cascade still exist [62, 63].

These gaps may be both patient- and practitioner-related. The most common patient-related barriers to treatment initiation include contraindications because of the presence comorbidities, lack of acceptance, and access of treatment, due to often asymptomatic course of the infection, long treatment duration and possible adverse reactions of the treatment, high cost, and distance to specialist [64–66]. In particular, the frequency and the severity of side effects related to pegylated interferon and ribavirin (PEG-INF/RBV) combination represent primary factors affecting both the initiation of therapy and its continuation. The tolerability of HCV therapy remained low also with the addition of first generation DAAs (telaprevir and boceprevir) to the above-mentioned therapeutic regimen [66]. The availability of new generation DAAs has improved the tolerability of the antiviral therapy [67].

Moreover, the linkage to care is critical among patients coinfected with HIV [68, 69], those with underlying psychiatric and substance use disorders, and those with social instabilities such as homelessness and incarceration [70].

General practitioner-related barriers include lack of expertise in HCV treatment, lack of specialty referral resources, resistance to treating persons currently using illicit drugs or alcohol, and concern about cost of HCV treatment [71–73].

Evidence-based interventions to address linkage to care have been summarized by Meyer JP and colleagues [61] and by the HCV Guidance by the Infectious Diseases Society of America (IDSA) and the American Association for the study of liver diseases (AASLD) recommended that at least annual HCV testing is in these subgroups [35].

These interventions include the integration of HCV screening, evaluation, and treatment center with other medical or social services, such as correctional facilities and programs providing needle exchange, substance abuse treatment, and methadone maintenance [74–76]. Multidisciplinary case management and social support have been demonstrated to facilitate the efficacious treatment of HCV-infected patients with psychiatric illness or substance use [77].

In order to address lack of access to specialists, models involving close collaboration between primary care practitioners and subspecialists also through telemedicine and multidisciplinary networks of specialists have been implemented [65, 78, 79]. Furthermore, the decreased duration and better safety profile of current HCV therapy may increase the number of mid-level practitioners and primary care physicians able to appropriately manage and treat HCV infection [35].

Additional strategies for improving linkage to and retention in care could be the use of patient navigators or care coordinators [80, 81]; however, the efficacy and effectiveness assessment of these interventions is still ongoing.

The extension of best and new strategies for linkage to HCV care is essential to optimize the impact of HCV treatment.

## 3. Do We Need DAAs Resistance Testing in the Future?

Treatment of chronic hepatitis C has rapidly evolved from PEG-INF/RBV to more potent highly effective DAAs combination therapies. In particular, DAAs act on key stages of the HCV lifecycle and are classified on the basis of their molecular target and mechanism of action [82]. Four categories of DAAs are currently available: NS3/4A protease inhibitors, NS5A inhibitors, nucleotide analogue inhibitors of NS5B RNA-dependent RNA polymerase (RdRp), and nonnucleoside inhibitors of RdRp.  Table 1 summarizes DAAs approved for HCV treatment. DAAs combination with other DAAs and/or ribavirin has been widely investigated in clinical trials and current recommendation by the World Health Organization (WHO) for the treatment of HCV with DAAs-based combination therapies is outlined in Table 2 [16]. During 2016, two novel DAAs combinations, grazoprevir/elbasvir and sofosbuvir/velpatasvir, have been licensed in US and Europe, and others are currently under evaluation in phases II and III clinical trials and may be available in the coming months [83].

The efficacy of treatment containing DAAs is very high in terms of sustained virological response (SVR); nonetheless 10–15% of therapeutic failure is observed in clinical practice, mainly associated with the selection of DAAs-resistant viral variants, resulting from mutations produced by amino acid substitutions in the target virus protein that reduce viral sensitivity to DAAs [84]. Resistance-associated variants (RAVs) may be present, despite being usually at low levels even before the beginning of DAAs treatment due to the great genetic variability of HCV [84]. Moreover, genotype 3 virus consistently demonstrates lower SVR rates to DAAs, despite higher SVR rates in other forms of the virus [85].

Since DAAs are widely used for treatment of HCV, the role of resistance-associated variants (RAVs) is becoming clearer.

Natural polymorphisms, before treatment, associated with resistance to NS3/4A, NS5A, and NS5B inhibitors have a considerable prevalence in DAAs naïve patients that is variable and depends on HCV genotype and subtype [86]. These variants may be selected rapidly during treatment with DAAs with the possible consequences of a viral breakthrough and treatment failure [86–88]. For example, the preexisting NS3/4A Q80K, mainly found in patients with HCV genotype 1a (5%–48%), reduces SVR rates in genotype 1a infected patients treated with the protease inhibitor simeprevir in combination with PEG-INF/RBV in comparison to those without Q80K [89, 90]. In phase III clinical trials of simeprevir and PEG-INF/RBV HCV genotype 1 and genotype 4 infected treatment-naïve patients and prior relapsers achieved SVR at week 12 rates of approximately 80% [91–95]. Response rates to therapy were lower in HCV genotype 1 patients with Q80K compared with HCV genotype 1 patients without this polymorphism [7, 9, 11]. The Q80K polymorphism is frequently observed in HCV genotype 1a [96]; meanwhile the occurrence is close to zero in HCV genotype 1b with the only exception of France where 11% of genotype 1b has Q80K [97, 98]. On the basis of these data, the European Medical Agency (EMA) and the Food and Drug Administration (FDA) strongly recommend testing for the presence of Q80K in HCV genotype 1a patients who are candidates to therapy with simeprevir and PEG-INF/RBV and discourage the use of this drug when Q80K is detected.

In the IFN-free regimen the presence of Q80K was associated with treatment failure only in a small sample size of patients with genotype 1a and cirrhosis: a phase 3 study (OPTIMIST-2) evaluated the efficacy and safety of 12 weeks of simeprevir plus sofosbuvir in HCV genotype 1 infected treatment-naïve or treatment-experienced patients with cirrhosis. Of the 72 patients with chronic HCV genotype 1a, SVR rate was lower for the 34 with Q80K at baseline than for the 38 without Q80K (74% versus 92%) [99]. For this reason, screening for the presence of the Q80K polymorphism should be considered before initiating simeprevir in combination with sofosbuvir in cirrhotic patients infected with HCV genotype 1a.

Reduced sensitivity to the first generation protease inhibitors (PIs) (boceprevir and telaprevir) and to the more recent PIs was associated with other NS3/4A mutations, such as those involving the amino acid positions V36, T54, R155, A156, and D168. The list of the major NS3/4A RAVs is reported in Table 3.

The RAVs conferring resistance to NS5A inhibitors more frequently occurred as natural variants in HCV G1 infected patients naïve to DAAs [100] and seemed to be more problematic in the setting of retreatment. Y93H is the most frequent baseline NS5A RAV in G1b, followed by L31M/V, while NS5A RAVs are less frequent in G1a [86, 87, 101–103]. L31M confers low-medium level resistance to daclatasvir and ledipasvir, while Y93H/N confers medium-high level resistance to all three approved NS5A inhibitors [100, 104] in G1a but only for ledipasvir in G1b.

Recently, the FDA recommended testing for the presence of virus with NS5A resistance-associated polymorphisms (substitutions at amino acid positions 28, 30, 31, or 93) in genotype 1a, regardless previous treatment history and cirrhosis status, before the combination therapy with grazoprevir and elbasvir to determine dosage regimen and duration (for 12 or 16 weeks and if ribavirin should be added) [101].

It is noteworthy that the persistence of NS5A RAVs has been observed in about 85% of patients with treatment failure over 1-2 years after the start of therapy [100, 105–107]. Therefore, testing polymorphism could be useful prior to retreatment decision because persistent NS5A RAVs could impact the second line therapy success [90]. The list of the major NS5A RAVs is reported in Table 4.

Regarding the NS5B RAVs, no cross resistance is observed across currently approved nucleotide and nonnucleoside polymerase inhibitors. Nucleos(t)ide inhibitors demonstrate activity against different HCV genotypes and have a high resistance barrier [97]. The principal mutation that confers decreased susceptibility to sofosbuvir is S282T and more recently the variants L159F (with/without L320F and C316N) and V321A were detected in patients with failed treatment [108]. M414T and S556G variants were observed in G1a and S556G in G1b patients who did not achieve an SVR after a nonnucleoside analog inhibitor dasabuvir-based regimen [104]. The most relevant NS5B RAVs are reported in Table 5.

Resistance testing at baseline and following treatment failure is not yet indicated, as more data are needed to demonstrate its role in clinical practice, but the unresolved issue is if it may be useful to individualize the best treatment option for each patient in the future. With the exception of Q80K and NS5A RAVs detection in G1a patients before starting simeprevir plus PEG-INF/RBV and grazoprevir plus elbasvir, respectively, whether resistance mutation testing should be done in treatment-naïve patients remains controversial.

Some authors highlight the importance of resistance testing in patients who fail multiple DAAs for deciding retreatment and selecting salvage therapy [90, 108, 109]. More recently, Sarrazin encourages also the baseline testing in patients treated with a combination of a first generation NS3/4A protease and NS5A inhibitor with a low barrier of resistance. Instead, in case of treatment with high antiviral activities and high genetic barrier to resistance, the evaluation of additional predictors of response is to be assessed. Moreover, baseline resistance testing may be used to choose the best DAAs regimen in patients with shortened treatment duration or with liver cirrhosis. Sarrazin sustained that in the future it is to evaluate if baseline testing could be cost-effective in order to prevent nonresponse to very expensive treatment, particularly in regions with economical restriction [100].

## 4. How Close Is an Effective Preventive HCV Vaccine?

The development of an effective HCV vaccine is hampered by several factors. First, HCV is characterized by an extraordinary genetic variability resulting from the lack of proofreading activity of the NS5B RNA-dependent polymerase [110]. This determines an impressive error rate per replication cycle that, in combination with the short viral half-life and the rapid turnover, leads to the generation of multiple distinct but closely related HCV variants, known as “quasispecies,” in one infected subject [111, 112]. Mutated viruses have the ability to persist in infected people by escaping immune control of cytotoxic T lymphocytes (CTL) and antibodies against different regions of the viral envelope [113, 114].

In addition to evading antibody and CTL recognition by passively mutating its genome in response to immune pressure, HCV exploits several further strategies to escape adaptive immune response and achieve a high rate of chronic infection. In fact, HCV, through a yet-unknown mechanism, fails to properly initiate the CD4+ T cell response at the beginning of infection and determines a rapid immune exhaustion and depletion of CD8+ T cells [115–117]. Moreover, HCV is able to hide from humoral immunity and pass from cell to cell without being exposed to the circulating antibodies by (i) binding low density lipoproteins, thus limiting the production of neutralizing antibodies during acute infection, (ii) decreasing viral immunogenicity through the presence of three glycans at the CD81 binding site of E2 glycoprotein, (iii) infecting surrounding cells through cell-to-cell contact mediated by CD81 and Claudin-1, and (iv) inducing interfering antibodies by constant mutation [118].

The weaker and ineffective adaptive immune response to HCV mainly contributes to the infection progressing into a chronic state. On the other hand, these immune evasion mechanisms have relevant implications in the development of the HCV vaccine. In fact, an effective vaccine should be able to induce strong neutralizing antibodies as well as powerful cellular immune responses during the very first stages of the HCV infection, before the virus has the chance to activate its many immune escape mechanisms [119].

A further barrier that has challenged the research on HCV vaccine is the lack of convenient experimental model systems for the study of HCV pathogenesis and vaccine design. To date, the only suitable infectious animal model is the chimpanzee. However, some ethical issues, the high cost of acquiring and maintaining the chimpanzees, and their limited supply restricted the use of these animals and the statistical power of studies [8, 120]. Furthermore, immune response of chimpanzees to HCV greatly differs from humans. Therefore, immunological results obtained with this model should be prudently interpreted [121, 122].

Several strategies have been adopted to develop an effective preventive HCV vaccine. They ranged from the traditional strategy of producing recombinant envelope proteins combining them with adjuvant substances to complex manufacturing of viral vectors directing the expression of multiple viral antigens. All strategies have been targeted to enhance the T cell response and, in particular, to determine a long-lasting cellular immune responses involving helper CD4+ and CD8+ T cells rather than only improving humoral responses.

Despite two decades of research efforts, few HCV candidate vaccines have reached the clinical trials phase and the evidences on both the efficacy and safety of these vaccines in humans remain limited (Table 6).

The first prophylactic HCV vaccine tested in human beings was a C-terminally shortened recombinant E1 protein adjuvant with aluminum hydroxide, called T2S-918/InnoVac-C, demonstrating good tolerability and good antibody response against E1. Moreover, a robust specific cellular immune response towards E1 was stimulated in all vaccinees [123]. However, the studies on this candidate vaccine were stopped in 2007.

Further HCV prophylactic vaccines based on recombinant proteins combined with adjuvant substances have been developed and evaluated in clinical trials.

A recombinant E1/E2 heterodimer vaccine (derived from HCV 1a) adjuvant with MF59C was tested in phase I clinical trial involving 60 healthy subjects. Vaccine demonstrated inducing neutralizing antibodies and T cell responses to E1/E2 in all subjects. Although the vaccine was safe and well-tolerated, its usage was prevented by manufacturing difficulties [124].

Another approach, based on recombinant protein, consisted of combining recombinant HCV core protein with an adjuvant substance, called ISCOMATRIX™. In a phase I clinical trial, conducted on 30 healthy volunteers, all subjects except one demonstrated antibodies against HCV core protein, without indication of a dose response. However, T cell responses were detected in only two subjects who received the highest dose of vaccine. The candidate vaccine was generally well-tolerated [125].

Also the development strategies based on HCV peptides vaccine that were able to induce HCV specific T cell responses by presenting vaccine peptide to the T cell receptor via HLA molecules have obtained some encouraging findings.

In particular, a peptide vaccine, known as IC41 and consisting of five synthetic peptides derived from conserved regions of core, NS3 and NS4 proteins of HCV genotypes 1 and 2 with a poly-L-arginine adjuvant, was evaluated in 128 healthy volunteers in a phase I clinical trial where it resulted in being safe and well-tolerated [126]. On the other hand, IC41 elicited few interferon-producing cells and dose-dependent T cell immune responses, even though a correlation between higher responder rates with dose and number of vaccinations was demonstrated [127]. In a successive randomized clinical trial, 54 healthy subjects received either subcutaneous or intradermal IC41 vaccine weekly (16 injections) or every other week (8 injections). One group additionally received imiquimod, an activator of the toll-like receptor (TLR) 7. Results showed that IC41 induced significant immunological responses in all groups with responder rates of up to 100%, even though imiquimod was not able to increase immunogenicity but was associated with a lower immune response. Intradermal injections caused more pronounced reactions, especially erythema and edema, but immunization with IC41 resulted generally in being safe and well-tolerated [127].

More innovative strategies to develop an effective HCV preventive vaccine include the manufacturing of DNA vaccine and viral vectors expressing HCV genes. DNA vaccines showed the capability of inducing cytotoxic lymphocyte responses in animal models; however, the induced immunity is often brief, weak, and unlikely to be effective in infection prevention. Viral vectors able to express foreign antigens represent an effective tool to induce a broader CD4+ and CD8+ T cell responses, compared to peptide vaccines.

Two preventive vaccines based on human adenovirus rare serotype 6 (HADV6) and chimpanzee Ad 3 (ChAd3) expressing the HCV nonstructural proteins were tested in a phase I clinical trial conducted on 36 healthy volunteers. The study demonstrated that both vaccines induced by specific T cell responses against multiple HCV proteins and T cell were capable of recognizing heterologous HCV strains (genotypes 1a and 3a). These data suggested that an adenoviral vector strategy may induce sustained T cell responses of a magnitude and quality associated with protective immunity, thus encouraging studies of novel prophylactic vaccines for HCV [128].

On the basis of some promising data obtained in phase I clinical trials, different types of prophylactic HCV vaccine are approaching phase II and phase III clinical trials [129]. In the next future, research efforts should be focused on improving the selection of the viral component and the proper administration regimens as well as on the safety and the tolerability of candidate preventive HCV vaccines.

## 5. Further Therapeutic Options

Research efforts for the development of alternative therapeutic options have been made contextually to the development of DAAs and the research on preventive vaccines. To date, several approaches have been adopted in the development and production of HCV therapeutic vaccines including peptide vaccines, recombinant protein vaccines, DNA vaccines with different carriers, and virally vectored vaccines.

Some studies were conducted among chronic hepatitis C patients, obtaining promising results in terms of T cell proliferation and IFN-*γ* responses and SVR rates within patients treated with interferon [130, 131].

Clinical trials, conducted by administering candidate HCV therapeutic vaccines in HCV chronic infected patients, have demonstrated HCV specific immune responses and transiently reduction of viral RNA, but the vaccines were not able to completely clear HCV infection or consistently reduce viral titers [130, 132, 133].

Some authors discussed the possibility to improve the efficacy of these vaccines by prior treatment with DAAs to first suppress HCV viremia [9], but the promising results obtained with the new generation of DAAs and their combination in curing HCV patients may overcome the use of therapeutic vaccination as an effective strategy for HCV treatment.

A further therapeutic option to cure HCV is represented by neutralizing monoclonal antibodies (mAbs) directed against HCV envelope glycoproteins E1 and E2, which are involved in the HCV entry into host cells or against phospholipids expressed on infected host cells [134, 135]. With respect to mAbs directed against viral antigens, due to the low immunogenicity of E1 glycoprotein, the research mainly focused on mAbs targeted conserved regions of E2 glycoprotein. To date, two mAbs directed against this target have been investigated in phase I and phase II clinical trials in the prevention of HCV reinfection during and after liver transplantation.

Two studies involved anti-HCV/2 human mAb HCV-AB68 demonstrating only a modest and short-lasting reduction in viremia [136, 137]. More recently, MBL-HCV1 showed good tolerability, reduced viral load, and delayed median time to viral rebound compared to placebo treatment in a phase II clinical trial involving 11 patients infected with HCV genotype 1a [138].

As regards mAbs against host receptors, a phase II unpublished clinical trial involving bavituximab was conducted to study the safety profile and early virological response in HCV genotype 1 infected patients treated with this mAb in combination with ribavirin in comparison with patients treated with PEG-INF/RBV [135].

In recent years, novel mAbs with immunomodulatory effects have been explored. Among these, mAbs that target programmed death 1 (PD-1), cytotoxic T lymphocyte-associated antigen 4 (CTLA-4), and CD3 receptors were studied in phase II clinical trials with encouraging results in terms of safety, tolerability, and viral response [139–141].

The most likely future for clinical use of mAbs is represented by regimen for HCV treatment consisting of mAb cocktails or combination of mAbs with other available drugs with the main goal of avoiding viral escape and the development of resistance.

## 6. Conclusions

Substantial advances have been made in HCV research and treatment in recent years. Although highly effective anti-HCV drugs are now available, these treatments remain poorly accessible because of their high costs; moreover, their efficacy is challenged by the high frequency of resistance-associated mutations. A further major obstacle for the control of the disease is represented by the lack of availability of an effective screening strategy to identify all people in need of treatment. Therefore, even though new drugs may contribute to impacting HCV spread worldwide, substantially modifying the natural history of the disease, eradication will be reached only through the development of an effective prophylactic vaccine. Future research directions should bridge this gap and progress in the comprehension of biological and immunological mechanisms of the disease and advances in molecular vaccinology, together with a strong financial, political, and societal support, will enable reaching this fundamental success in the coming years.

## Figures and Tables

**Table 1:** Direct-acting antivirals (DDAs) approved for HCV treatment or investigated in clinical trials (updated in September 2016).

Class	Generation	Approved substances (developing company)	Substances currently tested in clinical trials (developing company) [phase of development]
NS3/4A protease inhibitors	First generation	Telaprevir (Janssen, Mitsubishi) Boceprevir (Merck) Simeprevir (Janssen) Paritaprevir (AbbVie) Asunaprevir (Bristol-Myers Squibb) Vaniprevir (Merck)	
	Second generation	Grazoprevir (Merck)	ABT-493 (AbbVie) [Phase 3] GS-9857 (Gilead Sciences) [Phase 3]

NS5A inhibitors	First generation	Daclatasvir (Bristol-Myers Squibb) Ledipasvir (Gilead Sciences) Ombitasvir (AbbVie) Elbasvir (Merck) Velpatasvir (Gilead Sciences)	Odalasvir (Janssen) [Phase 2] Ravidasvir (Presidio) [Phase 2/3]
	Second generation		ABT-530 (AbbVie) [Phase 3] MK-8408 (Merck) [Phase 2]

Nucleotide analogue inhibitors of NS5B RNA-dependent RNA polymerase	First generation	Sofosbuvir (Gilead Sciences)	MK-3682 (Merck) [Phase 2] AL-335 (Janssen) [Phase 2]

Nonnucleoside inhibitors of NS5B RNA-dependent RNA polymerase	Palm-1 inhibitors	Dasabuvir (AbbVie)	

**Table 2:** Recommended regimen for the treatment of HCV with direct-acting antivirals-based combination therapies.

Genotype	Presence of cirrhosis	Recommended regimen	Alternative regimen	Strength of recommendation	Quality of evidence
1a	Without cirrhosis	DCV + SOF (12 weeks) LDV + SOF (12 weeks)^a^	SMV + SOF (12 weeks)^c^ OBV + PTV/r + DSV + R (12 weeks)	Strong	Moderate
		
	With cirrhosis	DCV + SOF (24 weeks) DCV + SOF + R (12 weeks) LDV + SOF (24 weeks) LDV + SOF + R (12 weeks)^b^	SMV + SOF (24 weeks)^c^ SMV + SOF + R (12 weeks)^c^ OBV + PTV/r + DSV + R (24 weeks)		

1b	Without cirrhosis	DCV + SOF (12 weeks) LDV + SOF (12 weeks)^a^	SMV + SOF (12 weeks) OBV + PTV/r + DSV (12 weeks)	Strong	Moderate
		
	With cirrhosis	DCV + SOF (12 weeks) LDV + SOF (12 weeks)	SMV + SOF (24 weeks) SMV + SOF + R (12 weeks) OBV + PTV/r + DSV + R (12 weeks)		

2	Without cirrhosis	SOF + R (12 weeks)	DCV + SOF (12 weeks)	Strong	Low
		
	With cirrhosis	SOF + R (16 weeks)	DCV + SOF (12 weeks)		

3	Without cirrhosis	DCV + SOF (12 weeks) SOF + R (24 weeks)		Strong	Low
		
	With cirrhosis	DCV + SOF + R (24 weeks)	SOF + PegIFN + R (12 weeks)		

4	Without cirrhosis	DCV + SOF (12 weeks) LDV + SOF (12 weeks)	SMV + SOF (12 weeks) OBV + PTV/r + R (12 weeks)	Strong	Moderate
		
	With cirrhosis	DCV + SOF (24 weeks) DCV + SOF + R (12 weeks) LDV + SOF (24 weeks) LDV + SOF + R (12 weeks)^b^	SMV + SOF (24 weeks) SMV + SOF + R (12 weeks)^c^ OBV + PTV/r + R (24 weeks)		

5	Without cirrhosis	LDV + SOF (12 weeks)	SOF + PegIFN + R (12 weeks)	Conditional	Very Low
		
	With cirrhosis	LDV + SOF (24 weeks) LDV + SOF + R (12 weeks)^b^	SOF + PegIFN + R (12 weeks)		

6	Without cirrhosis	LDV + SOF (12 weeks)	SOF + PegIFN + R (12 weeks)	Conditional	Very Low
		
	With cirrhosis	LDV + SOF (24 weeks) LDV + SOF + R (12 weeks)^b^	SOF + PegIFN + R (12 weeks)		

DCV: daclatasvir; LDV: ledipasvir; SMV: simeprevir; SOF: sofosbuvir; OBV: ombitasvir; PTV: paritaprevir; DSV: dasabuvir; R: ribavirin; r: ritonavir; PegIFN: pegylated interferon.

^a^Treatment may be shortened to 8 weeks in treatment-naïve persons without cirrhosis if their baseline HCV RNA level is below 6 million (6.8 log) IU/mL. The duration of treatment should be shortened with caution.

^b^If platelet count <75 × 103/*μ*L, then 24 weeks' treatment with ribavirin should be given.

^c^ If positive for the Q80K variant, a simeprevir/sofosbuvir regimen should not be chosen.

**Table 3:** Natural prevalence of NS3/4A inhibitor resistance-associated variants (RAVs) detected by population sequencing and mean fold change in resistance associated with RAVs.

Position	Variant	Resistant to	Natural prevalence in HCV genotype							Mean fold change in resistance compared to wild-type replicon						References
			1a	1b	2	3	4	5	6	Boceprevir	Telaprevir	Simeprevir	Asunaprevir	Paritaprevir	Vaniprevir	
V36	A/C/G	BOC/TVR/PTV	n.o.	n.o.	n.d.	n.d.	n.d.	n.d.	n.d.							[87, 142, 143]
V36	M	BOC/TVR	0.2–0.6%	0.1%	n.d.	n.d.	n.d.	n.d.	n.d.	3 (GT 1a)			2 (GT 1a)	2 (GT 1a)		[87, 142–147]
F43	I/L/S/V	SMV/ASV/PTV	n.o.	n.o.	n.d.	n.d.	n.d.	n.d.	n.d.							[86, 87, 148, 149]
T54	A	BOC/TVR	0.1–1.9%	n.o.	n.d.	n.d.	n.d.	n.d.	n.d.	2 (GT 1a) 3 (GT 1b)						[87, 144, 147, 150–153]
T54	S	BOC/TVR	0.4–3.1%	1.2–2.0%	n.d.	n.d.	n.d.	n.d.	n.d.							[87, 142, 143, 149]
V55	A	BOC/TVR	2.8%	0.4%	n.d.	n.d.	n.d.	n.d.	n.d.	3 (GT 1b)			1 (GT 1b)			[87, 145–147, 153]
Y56	H	PTV	n.o.	n.o.	n.d.	n.d.	n.d.	n.d.	n.d.							[86, 87, 144]
Q80	K	SMV/ASV/PTV	4.8–75.0%	0.5–1.2%	n.d.	n.d.	n.d.	n.d.	n.d.			11 (GT 1a) 8 (GT 1b)	3 (GT 1a) 1 (GT 1b)			[144, 146, 147, 154]
Q80	R	SMV/ASV	0.8%	0.6–0.7%	n.d.	n.d.	n.d.	n.d.	n.d.			6	1			[86, 87, 89, 144]
S122	R	SMV/ASV	n.o.	n.o.	n.d.	n.d.	n.d.	n.d.	n.d.			21				[86, 144, 150]
R155	K/I/G/L/M/T/Q/S	BOC/TVR/SMV/ASV/PTV	0.2–0.9%	n.o.	n.d.	n.d.	n.d.	n.d.	n.d.							[87, 142]
A156	F/N/S/T/V	BOC/TVR/SMV/ASV/PTV	n.o.	n.o.	n.d.	n.d.	n.d.	n.d.	n.d.							[86, 87, 89, 142, 148, 149]
V158	I	BOC	n.o.	0.1%	n.d.	n.d.	n.d.	n.d.	n.d.							[87, 143]
D168	E	SMV/ASV/PTV	0.2–0.3%	0.1–1.4%	n.d.	n.d.	n.d.	n.d.	n.d.			38 (GT 1b)	58 (GT 1a) 78 (GT 1b)	14 (GT 1a)		[86, 87, 89, 100, 142, 148, 149, 153, 155]
D168	G/H/V/TY	SMV/ASV/PTV	n.o.	n.o.	n.d.	n.d.	n.d.	n.d.	n.d.							[86, 87, 148]
V170	A	BOC/TVR	n.a.	0.1%	n.d.	n.d.	n.d.	n.d.	n.d.							[87, 142]
M175	L	BOC	n.a.	0.8–1.1%	n.d.	n.d.	n.d.	n.d.	n.d.							[87, 143]

BOC: boceprevir; TVR: telaprevir; SMV: simeprevir; ASV: asunaprevir; PTV: paritaprevir.

n.a.: not applicable because of different natural amino acid sequence in the respective HCV geno-/subtype (V170 and M175 are the dominant amino acids in GT1b).

n.o.: not observed.

n.d.: no data available.

GT: genotype.

**Table 4:** Natural prevalence of NS5A inhibitor resistance-associated variants (RAVs) detected by population sequencing and mean fold change in resistance associated with RAVs.

Position	Variant	Resistant to	Natural prevalence in HCV genotype							Mean fold change in resistance compared to wild-type replicon			References
			1a	1b	2	3	4	5	6	Daclatasvir	Ledipasvir	Ombitasvir	
K24	G/N	LDV	n.o.	n.a.	n.d.	n.d.	n.d.	n.d.	n.d.				[156]
K24	R	LDV	<1–1.5%	n.a.	n.d.	n.d.	n.d.	n.d.	n.d.				[156, 157]
M28	A	DCV/LDV	0.5%	n.a.	n.d.	n.d.	n.d.	n.d.	n.d.				[156]
M28	G	LDV	n.o.	n.a.	n.d.	n.d.	n.d.	n.d.	n.d.				[86, 156]
M28	T	DCV/LDV/OMV	0.4–1.8%	n.a.	n.d.	n.o.	82.0% (M28L)	n.d.	n.d.	205	61	8,965	[87, 100, 156, 158–160]
M28	V	OMV	3.5%	n.a.	n.d.	n.d.	n.d.	n.d.	n.d.			58 (GT 1a)	[86, 87, 100, 160–163]
F28	S	DCV	n.a.	n.a.	n.o.	n.d.	n.d.	n.d.	n.d.				[164]
L28	F/T	DCV/OMV	n.a.	n.o.	8.0%	n.d.	n.d.	n.d.	n.d.				[86, 87, 164]
Q30	H/R/E/L/T	DCV/LDV/OMV	0.3–1.3%	n.a.	n.d.	90.4–100%	50.0–100%	n.d.	n.d.				[86, 87, 156, 158, 165]
R30	H	DCV	n.a.	0.4%	n.d.	n.d.	n.o.	n.d.	n.d.				[87, 166]
R30	S	DCV	n.a.	n.a.	n.d.	n.d.	10%	n.d.	n.d.				[166]
R30	G/H	DCV	n.a.	n.a.	n.d.	n.d.	n.o.	n.d.	n.d.				[166]
A30	K	DCV	n.a.	n.a.	n.d.	2.3–6.3%	n.d.	n.d.	n.d.				[167]
L31	M	DCV/LDV	0.9–1.8%	2.1–6.3%	74.0–85.0%	1%	92.5–100%	n.d.	n.d.	105 (GT 1a) 3 (GT 1b)	554 (GT 1a)		[86, 87, 100, 145, 146, 151, 158, 160, 163, 164, 166–169]
L31	I/F/V	DCV/LDV/OMV	n.o.	0.7–1%	n.d.	n.o.	n.d.	n.d.	n.d.	15 (L31V)			[86, 87, 100, 156, 158, 159, 164, 166–168, 170]
P32	L	DCV/LDV	n.o.	<0.5%	n.d.	n.o.	n.o.	n.d.	n.d.				[86, 87, 100, 158]
S38	F	LDV	n.o.	n.o.	n.d.	n.d.	n.d.	n.d.	n.d.				[86, 100]
H58	D	DCV/LDV/OMV	<1%	n.a.	n.d.	n.d.	n.d.	n.d.	n.d.		1,127	243	[86, 87, 100, 144, 157]
P58	D	LDV	n.a.	n.o.	n.d.	n.d.	n.d.	n.d.	n.d.				[86, 100]
A92	K	LDV	n.o.	n.o.	n.d.	n.d.	n.d.	n.d.	n.d.				[86, 100]
A92	T	LDV	n.o.	2.8%	n.d.	n.d.	n.d.	n.d.	n.d.				[86, 100]
C92	R	DCV	n.a.	n.a.	n.o.	n.d.	n.d.	n.d.	n.d.				[164]
Y93	C/F/N	DCV/LDV/OMV	n.o.–0.6%	n.o.–0.7%	n.d.	n.o.	n.d.	n.d.	n.d.				[86, 87, 100, 158]
Y93	H	DCV/LDV/OMV	<1.5%	3.8%–14.1%	n.o.	1.3–8.3%	5–13%	n.d.	n.d.	12		77	[86, 87, 100, 156, 158, 159, 164–167, 169–175]
Y93	S	LDV	<0.5%	<0.5%	n.d.	n.d.	n.d.	n.d.	n.d.				[156]

DCV: daclatasvir; LDV: ledipasvir; OBV: ombitasvir.

n.a.: not applicable because of different natural amino acid sequence in the respective HCV geno-/subtype (K24, M28, Q30, and H58 are the dominant amino acids in GT1a; F28 is the dominant amino acid in subtype 2a and L28 in subtype 2b; A30 is the dominant amino acid in GT3).

n.o.: not observed.

n.d.: no data available.

GT: genotype.

**Table 5:** Natural prevalence of NS5B nucleotide and nonnucleoside inhibitor resistance-associated variants (RAVs) detected by population sequencing and mean fold change in resistance associated with RAVs.

Position	Variant	Resistant to	Natural prevalence in HCV genotype							Mean fold change in resistance compared to wild-type replicon		References
			1a	1b	2	3	4	5	6	Sofosbuvir	Dasabuvir	
S28	2T	SOF	n.o.	n.o.	n.o.	n.o.	n.d.	n.d.	n.d.	16		[87, 142, 176–180]
M289	I/L	SOF	n.o.	1.8%	3.5%	n.d.	n.d.	n.d.	n.d.			[176]
C316	Y	DSV	0.2–1.2%	n.o.	n.d.	n.d.	n.d.	n.d.	n.d.		1472 (GT 1a) 1569 (GT 1b)	[86, 87, 100, 165, 175]
C316	N	DSV/SOF	n.o.	10.9–35.6%	n.d.	n.d.	7.9%	n.d.	n.d.			[86, 87, 165, 181]
C316	H	DSV/SOF	n.o.	1.9–2.1%	n.d.	n.d.	n.d.	n.d.	n.d.			[86, 165]
L320	F	SOF	n.o.	n.o.	n.d.	n.d.	n.d.	n.d.	n.d.			[176]
S368	T	DSV	n.o.	n.o.	n.d.	n.d.	n.d.	n.d.	n.d.			[86]
N411	S	DSV	n.o.	n.o.	n.d.	n.d.	n.d.	n.d.	n.d.			[86, 142]
M414	T	DSV	0.5%	0.4%	n.d.	n.d.	n.d.	n.d.	n.d.		32 (GT 1b)	[87, 100, 175, 182]
M414	I	DSV	n.o.	n.o.	n.d.	n.d.	n.d.	n.d.	n.d.			[87, 142]
E446	K/Q	DSV	n.o.	n.o.	n.d.	n.d.	n.d.	n.d.	n.d.			[165]
Y448	C	DSV	n.o.	n.o.	n.d.	n.d.	n.d.	n.d.	n.d.			[86, 87, 142]
Y448	H	DSV	0.2%	1.3%	n.d.	n.d.	n.d.	n.d.	n.d.		975 (GT 1a)	[87, 144]
A553	I/T/V	DSV	6%	n.o.	n.d.	n.d.	n.d.	n.d.	n.d.			[165]
G554	S/D	DSV	n.o.	n.o.	n.d.	n.d.	n.d.	n.d.	n.d.			[86, 87, 142]
S556	G	DSV	0.6–3.1%	7.0–16%	100%	100%	97%	n.d.	n.d.		30 (GT 1a) 11 (GT 1b)	[86, 163, 181, 182]
S556	N/R	DSV	0.6–1.2%	n.o.	n.d.	n.d.	n.d.	n.d.	n.d.			[86, 87, 182]
G558	R	DSV	n.o.	n.o.	n.d.	n.d.	n.d.	n.d.	n.d.			[165]
D559	G	DSV	n.o.	n.o.	n.d.	n.d.	n.d.	n.d.	n.d.			[86, 87, 142]
Y561	H	DSV	n.o.	n.o.	n.d.	n.d.	n.d.	n.d.	n.d.			[165]

SOF: sofosbuvir; DSV: dasabuvir.

n.o.: not observed.

n.d.: no data available.

GT: genotype.

**Table 6:** Preventive hepatitis C virus vaccine tested in clinical trials.

Type of vaccine	Viral component	Adjuvant	Phase of clinical trial	Study population	Year	Reference
Recombinant protein	Recombinant E1 protein	Aluminum hydroxide	I	20 healthy subjects	2004	[123]
	Recombinant E1 and E2 proteins	MF59	I	60 healthy subjects	2010	[124]
	Recombinant core protein	ISCOMATRIX	I	60 healthy subjects	2009	[125]

Peptide	Five synthetic peptides derived from conserved regions of core, NS3, and NS4 proteins of HCV genotypes 1 and 2 (IC41)	Poly-L-arginine	I	128 healthy subjects	2006	[126]
	Five synthetic peptides derived from conserved regions of core, NS3, and NS4 proteins of HCV genotypes 1 and 2 (IC41)	Poly-L-arginine	I	54 healthy subjects	2010	[127]

Virally vectored	Human adenovirus rare serotype 6 (HADV6) and chimpanzee Ad 3 (ChAd3) expressing the HCV nonstructural proteins	—	I	30 healthy subjects	2012	[128]
